# Facile Sol–Gel Synthesis of Graphene-Supported FeNi Nanocatalysts for Enhanced Oxygen Evolution Reaction

**DOI:** 10.1021/acsomega.5c07875

**Published:** 2025-10-15

**Authors:** Romuald Teguia Doumbi, João Medeiros Dantas Neto, Artur de Morais, Awilvhygon Misker Dantas Freitas, Felipe Bohn, Kaline Pagnan Furlan, Dachamir Hotza, Carlos A. Martínez-Huitle, Marcio Assolin Correa

**Affiliations:** † Postgraduate Program in Materials Science and Engineering, 28123 Federal University of Rio Grande of Norte (UFRN), 59078-970 Natal, RN, Brazil; ‡ Department of Physics, Federal University of Rio Grande of Norte (UFRN), 59078-900 Natal, RN, Brazil; § Institute for Applied Materials − Ceramic Materials and Technologies (IAM-KWT), 150232 Karlsruhe Institute of Technology (KIT), 76131 Karlsruhe, Germany; ∥ Postgraduate Program in Materials Science and Engineering (PGMAT), 28117 Federal University of Santa Catarina (UFSC), 88040-900 Florianópolis, SC, Brazil; ⊥ Renewable Energies and Environmental Sustainability Research Group, Institute of Chemistry, Federal University of Rio Grande do Norte (UFRN), Natal, RN 59078-970, Brazil

## Abstract

The challenge of utilizing green energy sources remains relevant nowadays, especially in developing new catalysts for hydrogen production through complete water decomposition. For this purpose, an electrocatalyst based on an Fe–Ni alloy supported by graphene was synthesized. The catalyst was prepared by using the sol–gel technique. Physicochemical characterization showed that the main crystalline phase was the FeNi alloy with an equimolar ratio. EDS showed that the elements were well distributed on the graphene matrix. Analyzing the structural properties of the catalysts revealed that the GFeNi 1:1.0 catalyst, which has the smallest average crystalline size and lowest lattice strain, exhibited superior catalytic activity due to its high density of exposed active sites. This promotes excellent mass and charge transport during oxygen evolution reaction kinetics. Consequently, the catalysts demonstrated an overpotential of 0.268 V vs RHE at 10 mA/cm^2^ and maintained good stability in an alkaline 1 M KOH medium. The GFeNi 1:1.0 electrocatalyst showed electrochemical efficiency comparable to that of recently reported catalysts in the literature. These findings advance our understanding of ferromagnetic alloy-based materials for electrolysis applications and other electrochemical devices and highlight the potential of graphene-supported systems synthesized by sol–gel routes as cost-effective alternatives to noble metals.

## Graphical abstract

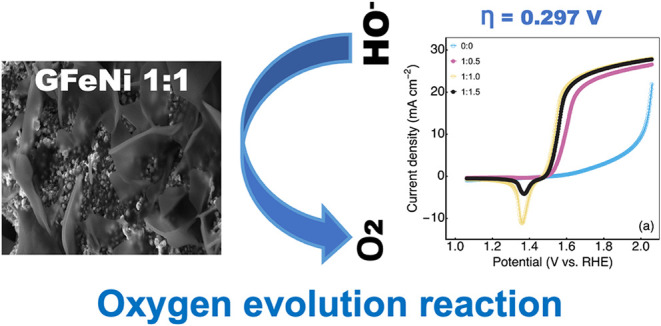

## 1 Introduction

Nowadays, the increasing demand for energy drives the search for alternative and environmentally friendly solutions for energy systems. Within this context, hydrogen has appeared as a promising candidate as a potential alternative fuel to replace fossil fuels.^1^ Hydrogen is already utilized in some industries and transportation systems, with its main advantage being the possibility to convert high amounts of energy with zero carbon dioxide emissions.^2^ However, not all forms of hydrogen generation are deemed environmentally friendly, which is mainly related to the carbon footprint of the method used to either directly synthesize or provide energy for the conversion. Consequently, depending on the method employed and the amount of carbon gases generated within it, hydrogen is classified in three main classes from least to most environmentally friendly: gray, blue, and green.^3^ Several methods are documented in the literature for the hydrogen generation or conversion,^4,5^ including gasification of natural gases,^6^ chemical reactions,^7^ and electrolysis.^5,8^ Among these techniques, electrolysis via electrochemical water splitting provides a considerably cleaner method, as it is able to generate pure hydrogen by using only electric current and an electrocatalyst. Especially the oxygen evolution reaction garners significant attention due to its high electrochemical potential (*E* = 1.23 V vs RHE) and the complexity of the reaction kinetics.^9,10^ Nevertheless, water electrolysis involves the evolution of oxygen at the anode and hydrogen at the cathode, which involves reactions requiring high overvoltage and, therefore, considerable energy input. Hence, there is a need to fabricate novel catalysts that are stable and efficient in enhancing the reaction kinetics and reducing the overvoltage.

The utilization of noble metals as electrode materials in electrochemical processes is still practiced due to their known and proven high electrocatalytic activity in reactions.^11^ However, the scarcity of these materials and their consequently high cost increasingly limit their widespread application.^12,13^ Thereby, numerous previous studies have focused on the investigation of alternate material systems with comparable electrochemical activity.^11,14^ Within this context, transition metals have been identified as a viable option for substituting noble metals, yet they are cost-effective and have a more abundant supply. Earlier reports by other authors have demonstrated the utilization of Fe and Ni for different electrochemical applications.^15–19^ Interestingly, these metals exhibit a variety of oxidation states: Fe (Fe^2+^, Fe^3+^, Fe^4+^) and Ni (Ni^2+^, Ni^3+^), enabling them to engage in complex redox mechanisms.^20^ Additionally, in alkaline media, these metals can form different oxides and hydroxides (Ni­(OH)_2_/NiOOH; FeOOH/Fe_2_O_3_), which are still active species for the oxygen evolution reaction (OER).^21–25^ Moreover, these metals have also demonstrated capability and activity for hydrogen evolution reaction (HER) and oxygen reduction reaction (ORR) in energy generation, conversion, or storage systems.^16,18,26–29^

Nonetheless, their electrocatalytic properties still fell short in comparison to noble metals, and thus, researchers have been investigating strategies to enhance their electrocatalytic properties. One of the strategies focuses on the functionalization of the transition metals with materials such as activated carbon, carbon nanotubes, graphene, and heteroatoms.^21,30–32^ Among these materials, graphene has garnered particular attention due to its excellent thermal and electrical conductivity, lightweight nature, flexibility, and large specific surface area.^13,33,34^ The utilization of graphene has been reported for energy storage, energy conversion using fuel cells, and hydrogen generation via electrolysis,^26,35–39^ where different properties are obtained depending on the fabrication technique. The most widely employed techniques include coprecipitation, hydrothermal synthesis, magnetron sputtering, solid-state synthesis, and sol–gel processes.^40–44^ In particular, the sol–gel technique is particularly favored for the synthesis of metal oxides due to its versatility and compositional control, as well as the capability for obtaining high-purity products, incorporating dopants, and generating hybrid materials.^41,45,46^

To date, only a few works have reported the application of the NiFe alloy and graphene-doped NiFe heterostructure for the electrochemical generation of oxygen gas. For example, in the work carried out by Ehsan et al.,^3^ the trimetallic catalyst Fe–Ni–V exhibited good performance for the OER activity with an overpotential of 370 mV at a current density of 1 A cm^–2^ and a durability of 40 h. These results were attributed to the spherical morphology of the as-prepared catalysts, which facilitates the connection of the catalysts with the highly conductive nickel foam used here as the support. Meanwhile, Shaopei et al. found an OER overpotential of 350 mV at a current density of 10 mA cm^–2^ using a Ni_12_P_5_–Ni_2_P heterojunction on graphene.^15^ Using the electrodeposition technique, Song et al., prepared a Co-doped Fe alloy, which presented an OER overvoltage of 268 mV at 10 mA cm^–2^.^1^ These previous studies demonstrated the great potential of Ni- and Fe-containing alloys or hybrids to improve the performance of the electrocatalysts, but a fundamental understanding of the influence of graphene in a FeNi alloy, forming a hybrid material system, is still to be explored. From the magnetic properties point of view, the NiFe alloy can present high magnetic permeability and very interesting soft magnetic properties, which can be easily manipulated by applying external magnetic fields,^47–49^ thus making the catalysts responsive to external stimuli. This unique characteristic of the FeNi alloy can improve the multifunctionality of the graphene-supported FeNi heterostructures.

While prior studies have demonstrated the promising activity of Fe- and Ni-based catalysts for various electrochemical reactions, the combined effect of graphene as a conductive support and FeNi alloys as the active phase remains insufficiently explored, particularly when synthesized via a sol–gel autocombustion procedure. Moreover, the influence of magnetic properties on the electrocatalytic behavior, enabled by the soft ferromagnetic nature of FeNi alloys, has not yet been systematically evaluated. To further understand and accurately determine the kinetic parameters of the catalysts with greater precision, electrochemical measurements were conducted with *iR* compensation.

Within this context, this work aims to address these gaps by investigating the structural, electrochemical, and magnetic characteristics of FeNi/graphene composites, thereby elucidating the synergy among material composition, morphology, and catalytic performance. Our findings demonstrated an overpotential of 0.268 V vs RHE at 10 mA/cm^2^ and maintained good stability in an alkaline 1 M KOH medium. In particular, the GFeNi 1:1.0 electrocatalyst exhibited electrochemical activity comparable to that of the state-of-the-art catalysts reported in the literature. These results advance the understanding of ferromagnetic alloy–based materials in electrolysis applications and further demonstrate the promise of graphene-supported systems synthesized via sol–gel methods as cost-effective alternatives to noble-metal catalysts.

## 2 Experimental Procedure

### 2.1 Materials and Synthesis

Graphene-supported FeNi powder was synthesized via sol–gel autocombustion using ferric nitrate Fe­(NO_3_)_3_·9H_2_O (99.96%), nickel nitrate (99.99%), citric acid C_6_H_8_O_7_ (99%), graphene nanoplatelets (99.99%), and sodium hydroxide NaOH (99.98%), which were purchased from Sigma-Aldrich. The method of synthesis of the catalysts is summarized in the scheme presented in Figure 1.

**Figure 1:**
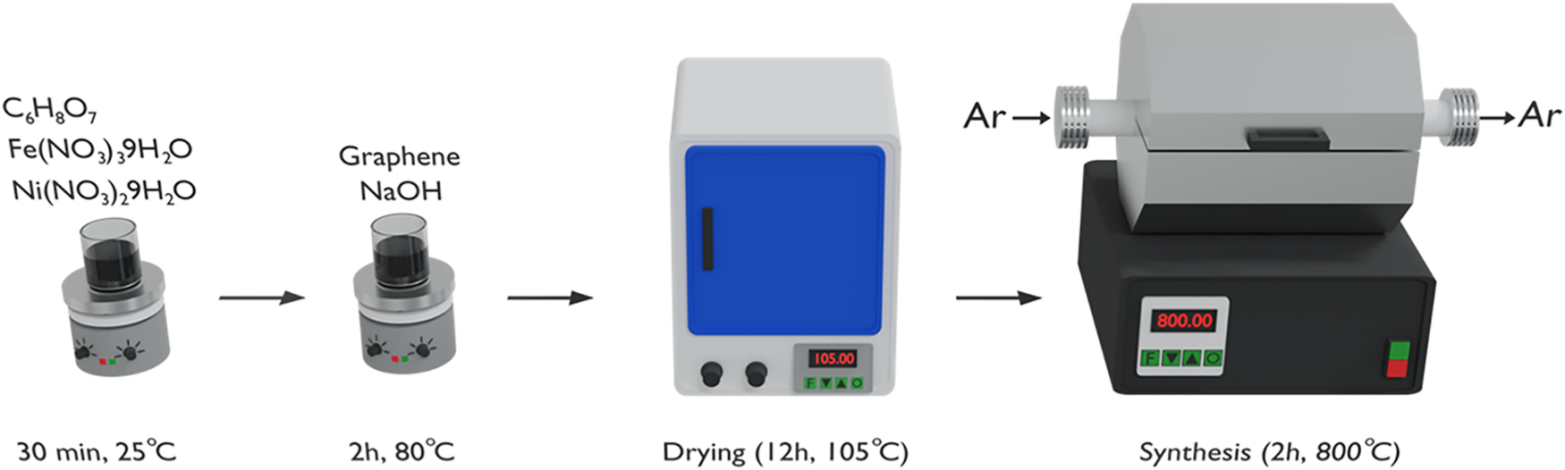
Schematic illustration of the synthesis procedure for GFeNi catalysts.

First, nickel nitrate (4.02 mg) and ferrous nitrate (8.04 mg) were dissolved in two volumetric flasks, resulting in an Fe:Ni ratio of 1:0.5. Next, citric acid (8.4 g) was dissolved in 40 mL of deionized water and stirred for 30 min. The previous solutions were then mixed and stirred for a further 30 min. Meanwhile, graphene nanoplatelets were dispersed in a solution of 5 M NaOH (10 mL) and 40 mL of deionized water for 15 min. Followingly, the nanoplatelets and the Fe:Ni solution were stirred for 2 h in a hot plate at 80 °C using a magnetic stirrer. After this period, the gel was collected and washed five times with deionized water, followed by drying in a furnace for 12 h at 105 °C. To consolidate the powder materials further, the material was sintered at 800 °C for 2 h in a tube furnace (FLYEYR-FE50RP) with an argon (Ar) atmosphere. The same procedure was used to fabricate samples with different Fe:Ni ratios of 1:1.0 and 1:1.5 by modifying the initial amount of nickel nitrate. In summary, 4 samples were investigated in this study: a bare graphene reference sample (GFeNi 0:0), and three graphene-supported FeNi catalysts with Fe:Ni molar ratios of 1:0.5, 1:1.0, and 1:1.5, respectively.

### 2.2 Characterization Techniques

Some physicochemical characterization techniques have been employed in this work to determine the properties of the as-synthesized materials. Thus, thermogravimetric (TG) and Differential Thermal analysis (DTA) were performed using Shimadzu DTG-60H equipment. An inert atmosphere was achieved by applying Argon (Ar) gas flow (50 mL/min), and the temperature range was set from 25 to 1000 °C with a heating rate of 7 °C/min. To investigate the formation and evolution of the phase structure, X-ray diffraction patterns (XRD, Rigaku Miniflex II diffractogram) were acquired using a Cu source (λ = 1.5406 Å) and Bragg–Brentano geometry. A step time of 2°/min with a 0.02° step size was employed in a 20 to 100° (2θ) range measurement. Scanning Electron Microscopy (SEM, Zeiss Auriga) was used to evaluate the samples’ morphology, while the energy-dispersive X-ray spectroscopy (EDX) module was used to assess the elements’ distribution and the chemical composition. Functional groups of the as-prepared catalysts were determined using Fourier Transform Infrared Spectroscopy (FTIR, Agilent 51420). Brunauer Emmet Teller method (BET, BELSORP Mini-II) was employed to evaluate the specific surface area of the catalysts. The magnetic properties of the samples were measured using a Physical Property Measurement System (PPMS) DynaCool from Quantum Design with a Vibrating Sample Magnetometer (VSM) modulus. The temperature varied from 5 to 300 K while an external magnetic field of ±14.0 T (140 kOe) was applied.

The electrochemical characterization was performed in a custom-built electrochemical cell consisting of a 1 M KOH support electrolyte and three electrodes immersed and connected to a potentiostat/galvanostat (Methrohm Autolab PGSTAT129 N). The three electrodes were a reference electrode (Ag/AgCl), a counter electrode (platinum wire), and a working electrode (the prepared catalyst). The working electrode was prepared from a homogeneous dispersion obtained by mixing 10 mg of catalyst with 225 μL of ethanol, 750 μL of distilled water, and 25 μL of Nafion solution (5%) to form a 10 mg-catalyst/mL mixture. The obtained suspension (0.3 mL) was deposited onto carbon felt (CF) (10 × 5 mm^2^) for the electrode preparation, leading to a loading of 6 mg cm^–2^. Then, the electrode was dried in a furnace for 4 h at 80 °C. The cyclic voltammetry (CV), linear sweep voltammetry (LSV), and electrochemical impedance spectroscopy (EIS) measurements were carried out under the above-described configuration. The 85% *iR* drop was used for *iR* correction between the reference electrode and the working electrode. This value was chosen based on the literature and employed in the same study.^50^ The uncompensated resistance was measured by performing an impedance measurement at high frequencies with a 10 mV sinusoidal amplitude at an open-circuit voltage (OCP). The measured electric resistance was 6.004 Ω. The LSV was carried out at a scan rate of 10 mV/s. EIS experiments were performed in the high-frequency range of 100 kHz to 0.1 Hz. All the measured potentials were converted to the reversible hydrogen electrode (RHE) from the following equation^3^

1 $$E\left(V\times \mathrm{RHE}\right)=E\left(Ag/AgCl\right)+E^{◦}\left(Ag/AgCl\right)+0.059pH$$

where *E*(Ag/AgCl) is the observed potential by using the Ag/AgCl (3 M) reference electrode, *E* ^o^(Ag/AgCl) is the standard potential of Ag/AgCl (3 M) equal to 0.197 V, and pH is the pH of the electrolyte.

## 3 Results and Discussion

### 3.1 Morphology and Elemental Distribution

The samples’ morphology reveals a highly porous and interconnected graphene network, characterized by distinct flake-like structures that serve as a matrix for the FeNi-based nanoparticles (Figure 2a,b). A closer look at Figure 2b confirms the intimate contact and uniform distribution of these nickel- and ferrous-based nanoparticles across the graphene sheets, indicating their successful integration. Furthermore, it is possible to observe the presence of porosity, which is generated during the autocombustion sol–gel synthesis and is particularly beneficial for applications in electrochemistry and energy storage, as it enhances ion diffusion and provides a large surface area for electrochemical reactions.^26^ This morphology is in agreement with previously reported structures synthesized via the autocombustion sol–gel method.^51^

**Figure 2:**
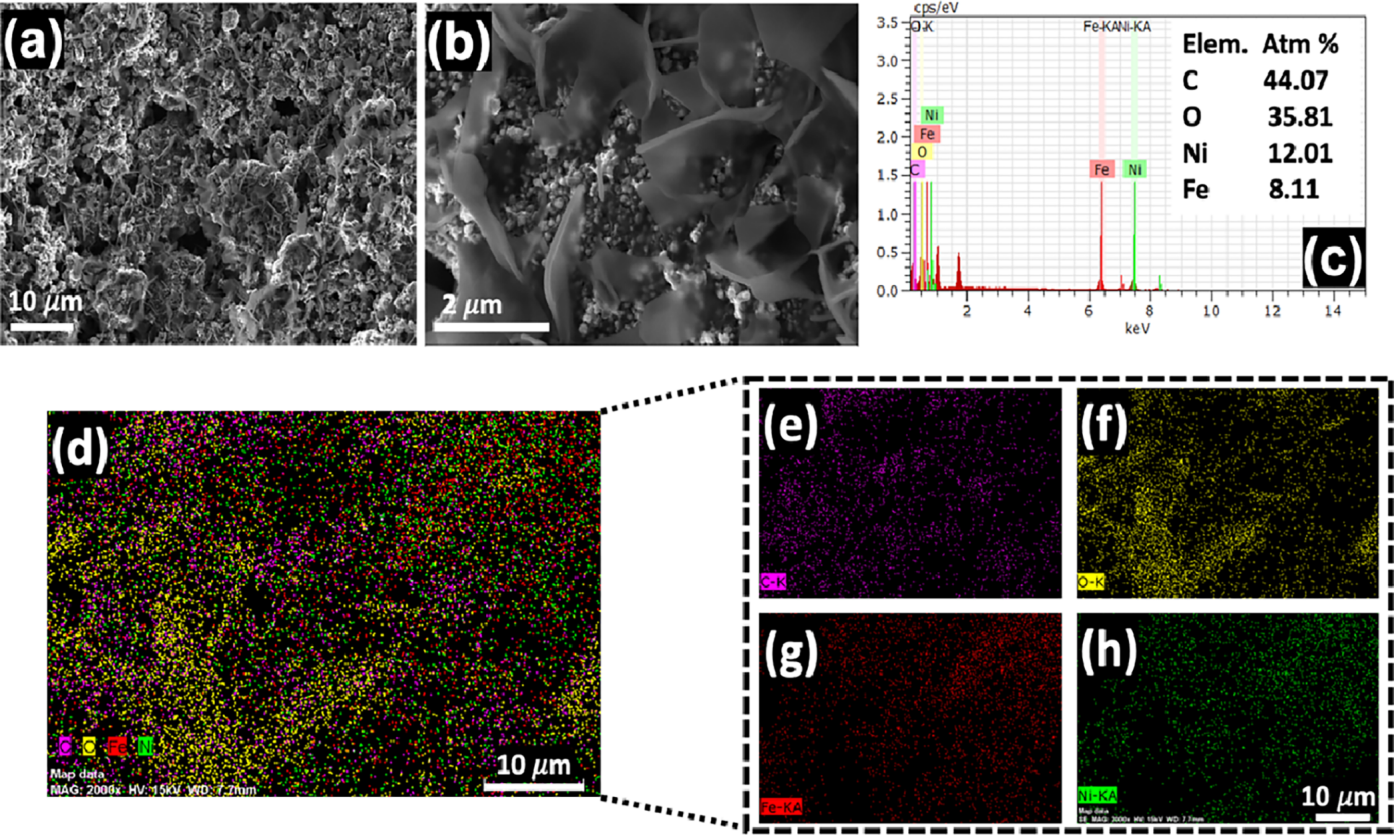
(a) SEM image of the GFeNi 1:1.0 sample with magnification of 2.0 K x, EHT = 5.00 kV. (b) SEM image of GFeNi 1:1.0 sample with magnification of 16.0 K X, EHT = 5.00 kV. (c) EDS results for the GFeNi 1:1.0 sample indicate the presence of the Ni and Fe elements on the material. (d) Color map of the elements (C, O, Fe, Ni). (e)-(h) Color map for each element in the studied material.

EDS measurements (Figure 2c) show the presence of carbon, iron, nickel, and oxygen as the elements. To verify the spatial distribution of each element in the sample, Figure 2d–h depicts the color map for each element present in the sample. From these figures, the elemental composition is comprised of Carbon (44.07%), Oxygen (35.81%), Nickel (12.01%), and Iron (8.11%). The high carbon content is associated with the graphene matrix, while the presence of nickel and iron attests to the successful incorporation of the metallic elements. Furthermore, elemental mapping demonstrates a homogeneous distribution of C, O, Fe, and Ni throughout the sample, confirming the effective dispersion of the Ni–Fe nanoparticles within the graphene matrix. This uniform dispersion of the Ni–Fe nanoparticles is expected to maximize the availability of active sites for the OER reaction, while promoting efficient charge transfer, which is crucial for the functional properties. The significant oxygen content is attributed to the oxide phase of the material, which is later discussed, and may further enhance catalytic activity by providing additional active sites.

### 3.2 Crystalline Structure and Phase Analysis

Figure 3a–d displays the X-ray diffraction (XRD) patterns for the samples prepared with varying iron-to-nickel molar ratios (GFeNi 1:0.5, 1:1.0, and 1:1.5) and the graphene reference sample. All samples exhibit a prominent peak at 26.55° attributed to the (002) plane associated with graphene, characteristic of the hexagonal graphite phase with a space group of P63mc corresponding to the crystallographic card ICSD 9012230. This result is consistent with the literature.^52^ Additionally, six main intensity peaks are observed at 2θ angles of 43.40, 43.90, 51.2, 75.56, 91.65, and 96.70°, which are assigned to the (111), (11–1), (200), (220), (131), and (222) planes of a FeNi alloy phase with a face-centered cubic crystalline structure phase fm3̅m (spatial group number 225) corresponding to the crystallographic card ICSD 103556. Moreover, other peaks are observed at around 35.70 and 63.10°, attributed to the (31–1) and (04–4) planes of a NiFe_2_O_4_ phase, space group fd3̅mS (ICSD 40040).^26^

**Figure 3:**
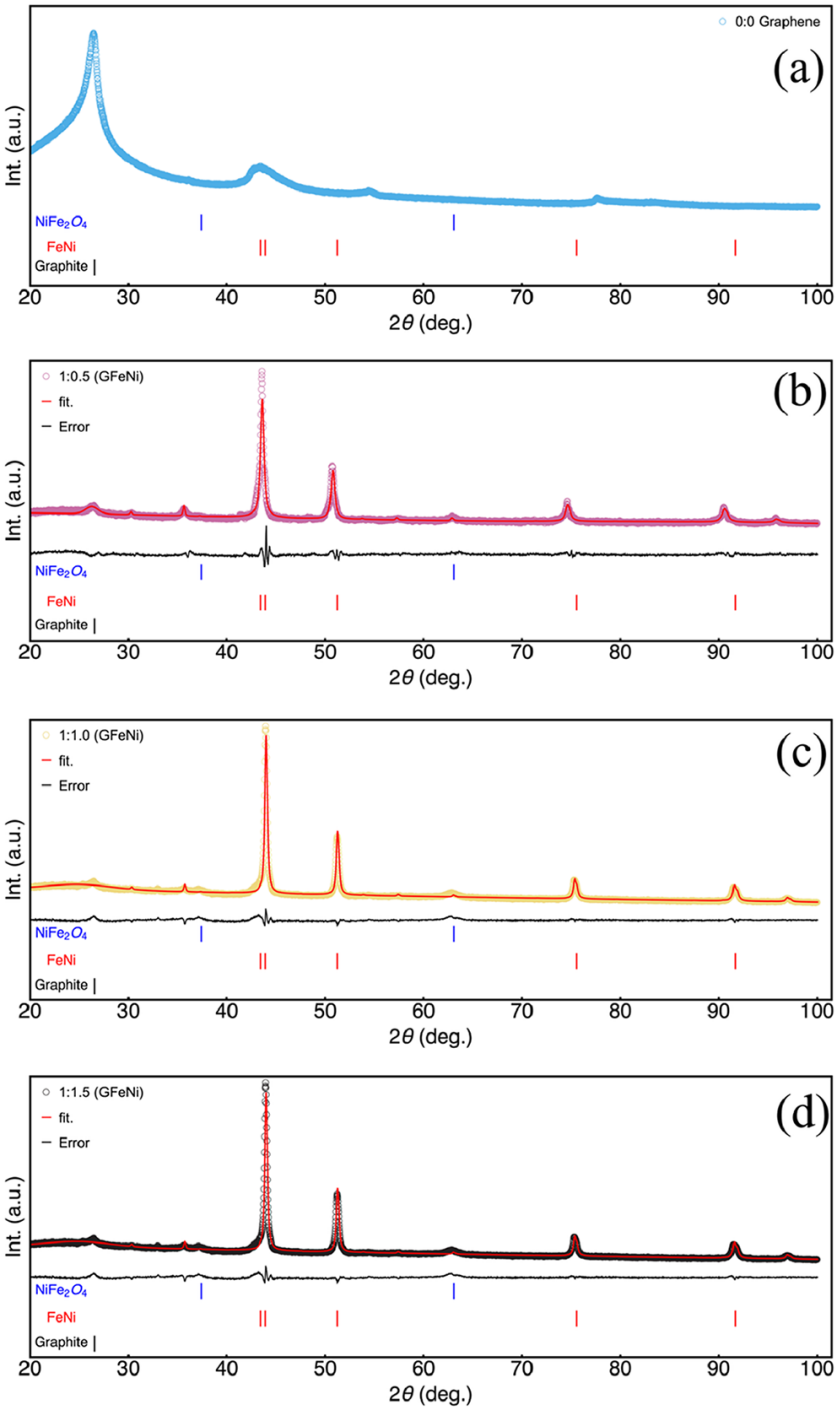
XRD pattern results for the prepared samples; the red line indicates the best fit realized by using Rietveld refinement. The black line indicates the error related to the Rietveld refinement. The peaks are indexed considering the cards ICSD 9012230, ICSD 103556, and ICSD 40040. (a) Results for the graphene sample. (b) Results for the GFeNi 1:0.5 sample. (c) Results for the GFeNi 1:1.10 sample. (d) Results for the GFeNi 1:1.15 sample.

For the sample with a lower amount of Ni (GFeNi 1:0.5), the diffractogram shows intense and well-defined peaks, indicating the formation of highly crystalline phases. The high intensity of these peaks also indicates that the FeNi alloy is this composition’s major and most crystalline phase. The main peak indicates that the cubic FeNi phase was well-indexed for this GFeNi sample. Furthermore, this suggests the effectiveness of the sol–gel autocombustion method for producing Fe:Ni-supported graphene catalysts. With an increase of the Ni content (GFeNi 1:1.0), the most prominent peaks are still associated with the cubic FeNi phase; however, subtle changes in relative intensities and peak broadening are observed. This suggests that while the cubic FeNi phase remains predominant at an equimolar Fe:Ni ratio, there might be minor modifications in its stoichiometry, the formation of structural defects, or the initial stage formation of an additional phase. The latter is confirmed in the sample with the highest nickel amount (GFeNi 1:1.5) sample, which presents a more complex diffractogram with multiple peaks of varying intensities, where the increased nickel content is hypothesized to favor the formation of a nonstoichiometric phase. To quantify the phases, Rietveld refinement was realized, and the results are summarized in Table 1.

**Table 1:** Rietveld Refinement Data for the Catalysts GFeNi 1:0.5, GFeNi 1:1.0, and GFeNi 1:1.5

			lattice parameters				quality of fit		
samples	phase	phase quantity (%)	*a*	*b*	*c*	crystallite size (nm)	χ^2^	*R*_wp_	*R*_exp_
GFeNi 1:0.5	FeNi	62.14	3.59	3.59	3.59	32.84	1.71	4.56	2.66
	C	33.62	2.53	2.53	6.78	94.43			
	NiFe_2_O_4_	4.24	8.33	8.33	8.33	99.989			
GFeNi 1:1.0	FeNi	60.26	3.56	3.56	3.56	53.03	2.24	3.26	1.45
	C	36.83	1.13	1.13	7.09	23.88			
	NiFe_2_O_4_	2.91	8.33	8.33	8.33	99.99			
GFeNi 1:1.5	FeNi	25.48	3.56	3.56	3.56	51.91	2.21	3.21	1.45
	C	73.30	1.82	1.82	7.09	92.6			
	NiFe_2_O_4_	1.22	8.33	8.33	8.33	99.99			

The Rietveld refinement of the XRD diffractograms shows that the peaks demonstrate asymmetry, and the quality of the refinements was significant based on the low values of χ^2^, *R*_wp_, and *R*_exp_. It was also observed that the increase in the Ni concentration favors the increase in the graphitic phase and the decrease in the NiFe_2_O_4_ phase. This observation has also been previously reported in the literature.^51^ This effect has a significant impact on the magnetic and catalytic properties, which are later discussed. Table 2 shows the average crystalline size and lattice strain. The average crystalline sizes of the as-prepared materials were 75.75, 58.97, and 81.5 nm, for GFeNi 1:0.5, GFeNi 1:1.0, and GFeNi 1:1.5, respectively. These low crystallite sizes are explained by the morphology of the catalyst (discussed previously, see Figure 2), where the graphene nanoplatelets network restricts the growth of the Fe:Ni-based phases. Furthermore, the lattice strain increases with the increase in the Ni content up to the concentration Fe:Ni 1:1.0 and then decreases. These results are associated with the formation of the oxide phase NiFe_2_O_4_, as observed from the refinement results, while the graphene leads to the mitigation of lattice strain and provides a more stable lattice arrangement, thus contributing to the reduction of the lattice strain observed for GFeNi 1:0.5.^51^ From the electrochemical point of view, the GFe:Ni 1:1.0 sample is expected to promote good electrochemical performance due to its high crystallinity, yet low average crystallite size and high lattice strain.

**Table 2:** Resumed the Average Crystalline Size and Microstrain of the Catalysts

catalysts	average crystalline size (nm)	microstrain (nm)
GFeNi 1 0.5	75.75	0.0077934
GFeNi 1 1	58.96	0.039227
GFeNi 1 1.5	81.51	0.035927

### 3.3 Functional Groups and Thermal Stability

Aside from the crystal structure, it is necessary to gain an understanding of the chemical structure, especially of the functional surface groups that might take part in the surface interactions and reactions. The FTIR spectra of the catalysts were presented in Figure 4a. The FTIR measurements of the different catalysts reveal the emergence of several absorption bands that increase with the increasing concentration of nickel content during synthesis. The stretching vibration band observed around 1350 cm^–1^ indicates the presence of C–O groups, as also observed in alcohols or ether oxides.^53^ Additional peaks at approximately 710, 650, and 600 cm^–1^ are characteristic of Fe–OH, Ni–O, and Fe–O vibrations, which confirms the formation of active hydroxide and oxide phases.^16,54^ Furthermore, the formation of Fe–OH, Ni–O, and Fe–O groups is deemed beneficial for the interaction with the electrolyte. These features indicate a surface structure that is adequate to be used as a catalyst for OER. Moreover, in the FTIR measurements of the reference graphene nanoplatelets (Figure 4a), it is possible to observe an absorption band around 2250 cm^–1^ characteristic of the vibration frequency of CO_2_ and attributed to the oxygen adsorption from the air. This band is also observed in the catalysts yet with lower intensity. The reduction in intensity of the CO_2_ absorption band peak, coupled with the absence of absorption bands for the C–H and OH functional groups in the FTIR spectra of the catalysts, suggests a modification of the surface properties due to the heat treatment in an argon atmosphere at 800 °C (final step of the synthesis).

**Figure 4:**
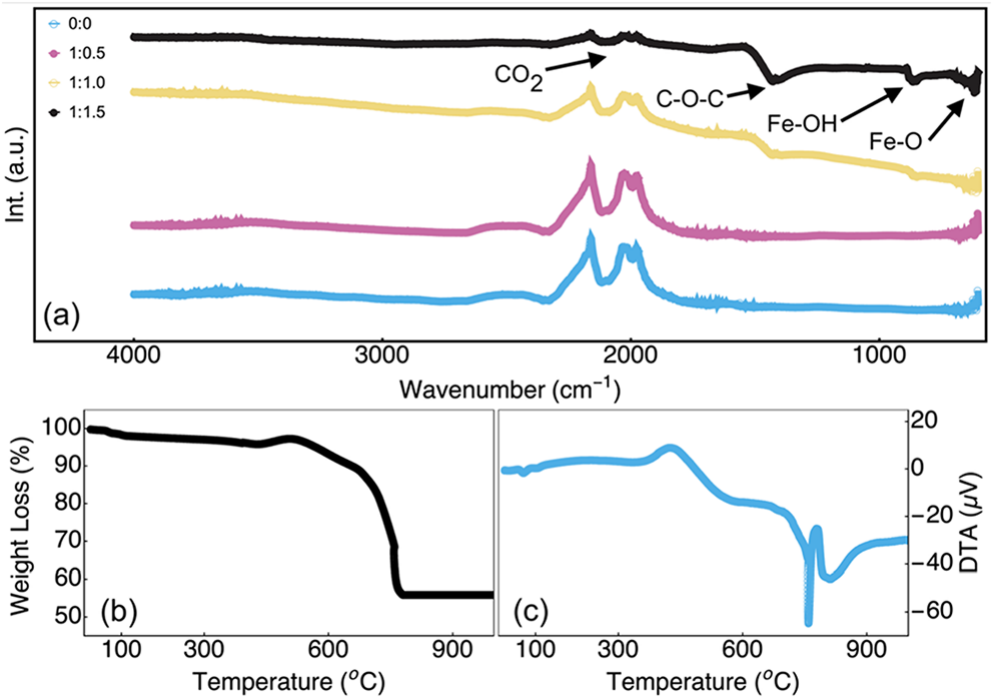
(a) FTIR curves for the graphene reference sample and the samples with different Fe:Ni ratios. (b) TG and (c) DTA results for the GFeNi 1:1.0 sample.

To further assess the behavior of the catalyst during the final step of the synthesis (autocombustion), TG-DTA measurement of the GFeNi 1:1.0 sample was performed under similar atmosphere and temperature conditions (Figure 4b,c). The results show a decomposition process divided into multiple steps. The first step indicates a significant weight loss (approximately 5%) occurring between 25 to 200 °C, attributed to the release of water molecules in the structure of the GFeNi powder. The second weight loss (2%) happens in the 200 to 430 °C range, and it is associated with the decomposition of the metallic nitrates.^26^ The third step starts at 430 °C and is characterized by an increase (5%) in the powder mass. This weight gain is possibly attributed to the oxide phase formation (NiFe_2_O_4_) with the oxygen coming either from residual oxygen within the atmosphere or within the powder upon the nitrate’s decomposition. However, during the synthesis of graphene-based catalysts, oxygen could be adsorbed on the surface, between layers, or trapped in the graphene structure. This oxygen, depending on its availability at high temperature, could react with carbon atoms or metals, leading to the formation of CO or metal oxide, responsible for the mass gain.^55–57^ The DTA results (Figure 4c) show an exothermic peak centered at 430 °C, supporting the oxygen-driven reaction hypothesis. Moreover, it also shows the presence of an endothermic peak centered at 98 °C, confirming residual absorbed water evaporation. The fourth decomposition step begins at around 550 °C, representing the major part (almost 40%) of the total weight loss. This phenomenon, related to a sharp, intense, endothermic peak, could be attributed to the decomposition of intermediated compounds like hydroxides and/or the transformation of the iron or nickel oxide phase to another.^10^ Finally, at 780 °C, the graph shows a straight line until the end of the analysis, displaying the stability of the weight loss and formation of the final product.

### 3.4 Magnetic Properties

Figure 5a depicts the magnetization curves of the samples measured at 300 K (room temperature) as a function of the external magnetic field, where ferromagnetic behavior is observed for all studied samples. Meanwhile, the pure graphene sample (0:0) presents room temperature ferromagnetism with a very low magnetic moment, as expected.^58,59^ Regarding the Fe:Ni samples, all curves exhibit a small coercive field and a low remanent magnetization. As the Fe:Ni content increases, an increase in the saturation magnetization *M*_s_ of the samples is observed (Figure 5b). Further measurements were performed in a reduced external magnetic field of ±14 kOe at 5 and 100 K (Figure 5c,d), demonstrating the stability of the magnetization behavior. These results attest to the success of the functionalization of ferromagnetic properties of the catalysts, which can be used as a feature to modify the oxygen evolution reaction as discussed below.

**Figure 5:**
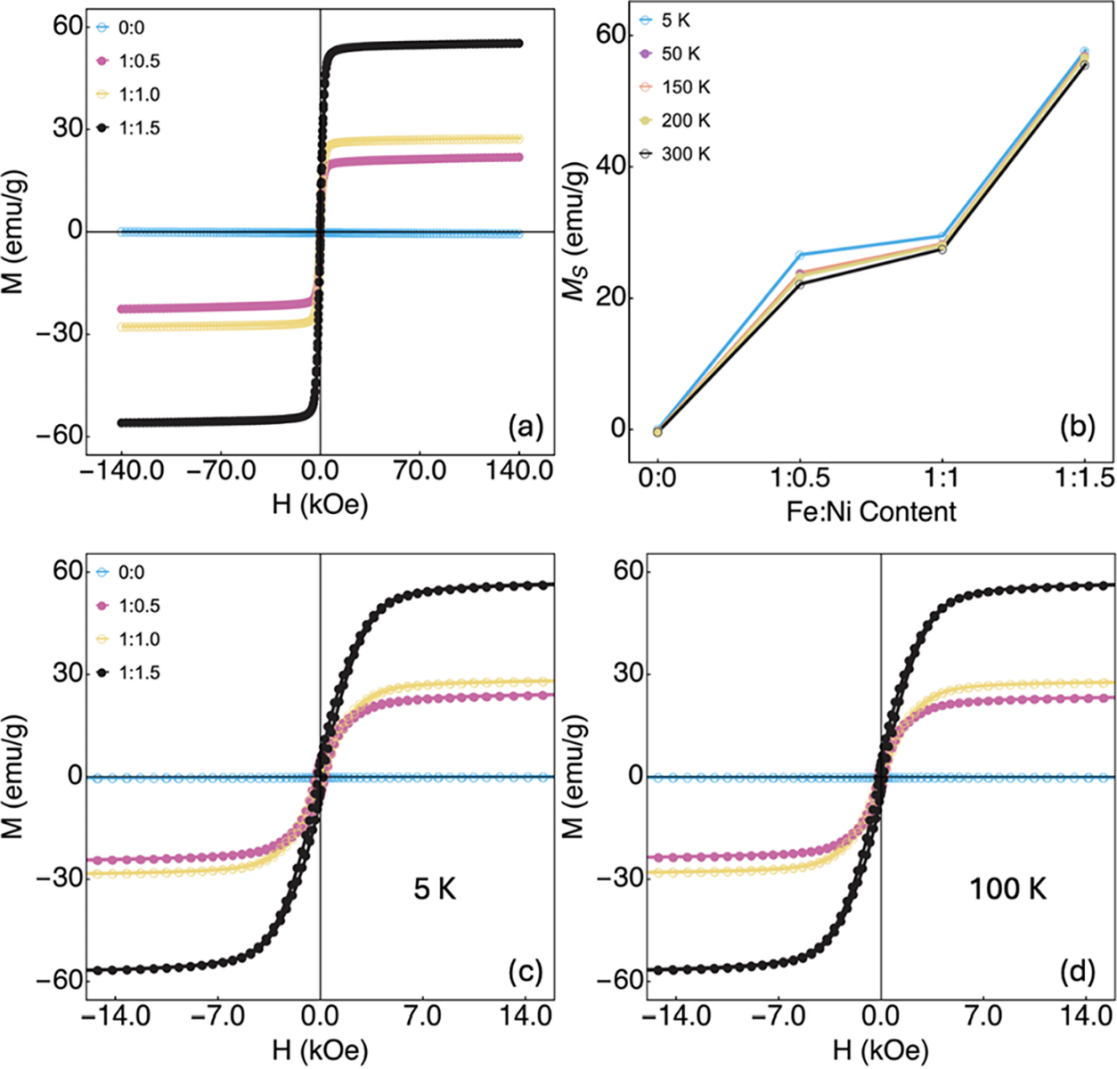
(a) Magnetization curves for a wide external magnetic field range (±140 kOe) measured at room temperature (300 K) for all study materials. (b) Saturation magnetization (*M*_s_) as a function of Fe:Ni content in the studied material, measured at a wide range of temperature (5 to 300 K). (c) Magnetization curves in a reduced external magnetic field range (±14 kOe) for all samples measured at 5 K. (d) Similar plot for the magnetization curves measured at 100 K.

### 3.5 Electrochemical Performance

The catalytic properties were assessed by measurement of the OER efficiency in a basic medium of 1 M KOH. In electrochemical measurements, the resistance of the solution could significantly affect the performance (applied voltage) of the system in the sense that it could be responsible for the voltage drops. Thus, the necessity of using the *iR* compensation during electrochemical tests is fundamental to measure with more precision the real potential implicated in the reaction and the accurate determination of the kinetic parameters. Cyclic voltammetry measurements were performed using a conventional three-electrode device in a 1 M KOH solution as the electrolyte, and the results are reported in Figure S1 (Supporting Information). Linear sweep voltammetry (LSV) measurements (Figure 6a) were also carried out to study the OER activity of the electrocatalysts. The onset potentials for hydroxide ion oxidation were observed at 1.675, 1.493, 1.472, and 1.451 V for electrocatalysts G, GFeNi 1:0.5, GFeNi 1:1.0, and GFeNi 1:1.5, respectively. Recently, it has been demonstrated that the catalyst performance could be influenced by the surface morphology. LSV curves plotted from the normalization current density by the electrochemically active surface area (ECSA) (Figure 6b) were used to compare the electrochemical performance of the catalyst by minimizing the effect of the surface area variation. The results revealed that the higher the ECSA, the lower the current density (mA cm^–2^ (ECSA)). Once again, the order of the OER onset potential was GFeNi 1:1.0, GFeNi 1:1.5, GFeNi 1:0.5, and G. Thus, the enhancement of the electrochemical performance is attributed to the increase in ECSA and not only to the intrinsic activity of Fe, Ni, and the graphene component. This result was also found in a previous study.^60^ Thus, the ECSA of the electrocatalysts has a significant influence on the electrochemical performance of the electrocatalysts. These results highlighted the real performances of each catalyst without the contribution of the ECSA.

**Figure 6:**
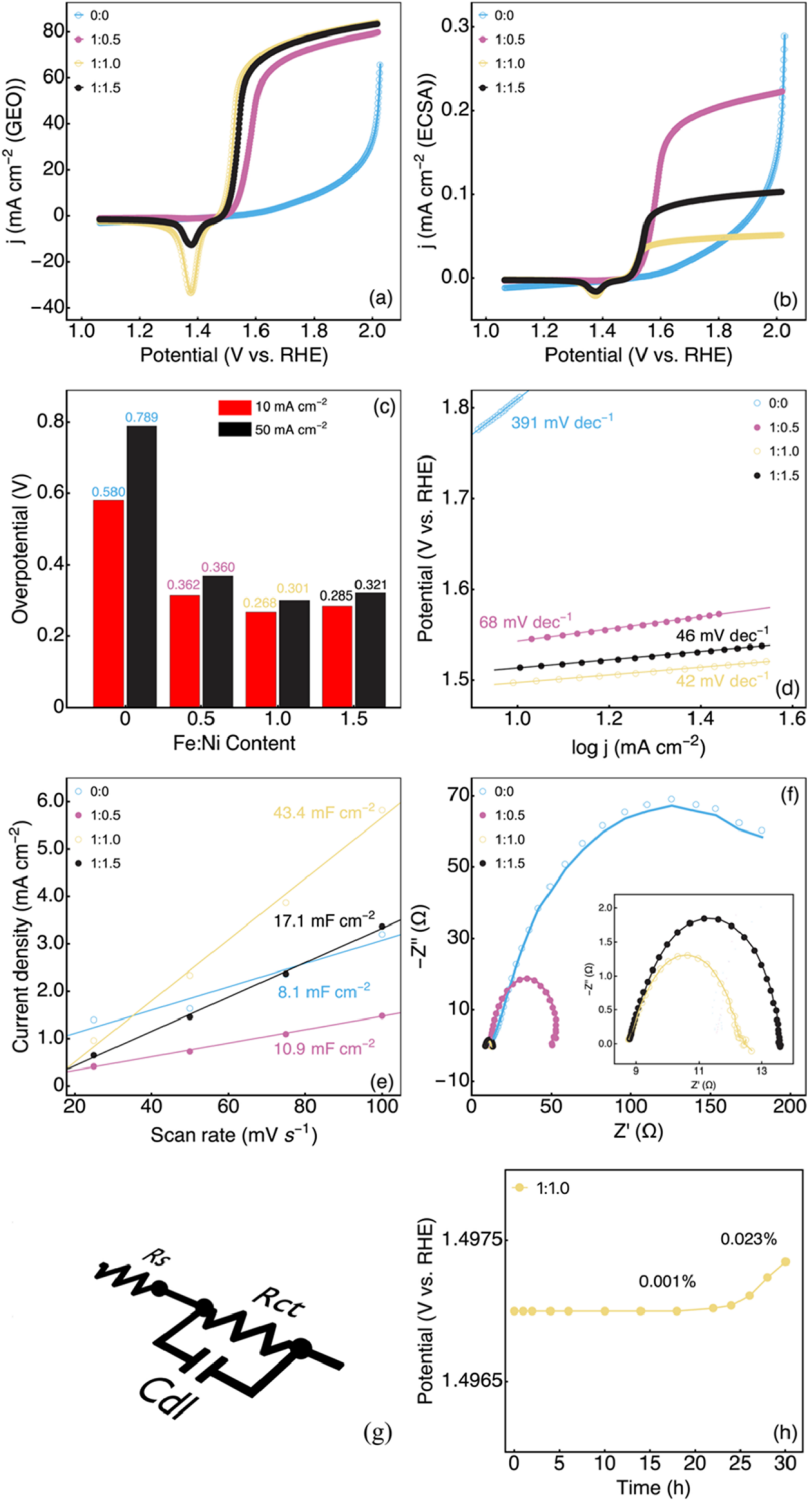
(a) LSV curves with *iR* correction normalized by geometric surface area. (b) LSV plots normalized by the estimated ECSA. (c) Oxygen overvoltages at 10 and 50 mA cm^–2^. (d) Tafel plots. (e) Calculated double-layer capacitance (*C*_dl_) used to evaluate the ECSA of the different catalysts. (f) Nyquist plots of the catalysts with the fitted curves. (g) Electrical equivalent circuit. (h) Long-term stability test.

Figure 6c illustrates the overpotentials required to achieve current densities of 10 and 50 mA cm^–2^. Among the samples tested, the GFeNi 1:1.0 catalyst exhibited the lowest overpotential, measured at 268 and 301 mV at 10 and 50 mA cm^–2^, respectively. This increased catalytic activity is attributed to the synergistic effects between graphene and the presence of NiOOH and FeOOH species on the catalyst surface, which likely optimize the binding energies of the intermediates at the electrode–electrolyte interface. However, the concentration of nickel during the catalyst preparation needs to be controlled because it influences the electrochemical performance of the system. This work highlights the importance of maintaining a balanced Fe/Ni ratio to achieve optimal OER efficiency. However, high concentrations of Ni could be responsible for the reduction of the performance of the electrode, by limiting the number of active sites and the charge transfer. It could also contribute to inhibiting the favorable free energy of intermediate states in the OER mechanism. This finding was also recently shown by Pan et al.^61^ when preparing Ni-based high-entropy alloys. The authors found that to achieve the best OER efficiency, the Ni percentage in the catalyst must be fixed at 50%; above this percentage, the efficiency decreased. The overpotential of GFeNi 1:1.0 is comparable to that of some of the most efficient iron-based OER catalysts described in the literature, as summarized in Table 3.

**Table 3:** Comparison of 3-d Transitional Metal-Based Electrocatalysts for OER

catalysts	synthetic route	electrolyte	Tafel slope (mV dec^‑1^)	OER Overp. (mV)	reference
NiFe	Sol gel	0.1 M KOH	153	320	^62^
Ir/CoNiS2	Chemical vapor deposition	1 M KOH	125	370	^63^
NG/Fe_2_TiO_5_	Chemical bath deposition	1 M KOH	35	264.2	^64^
Ni/NiO	Coprecipitation	1 M KOH	70	346	^65^
Co–Fe	Electrodeposition	1 M KOH	38.41	268	^1^
Ru_*x*_Fe_2_Ni_5_	Coprecipitation	1 M KOH	76	235	^66^
CoNiCuMnFe/C	Coprecipitation	1 M KOH	79	350	^67^
Ni_12_P_5_–Ni_2_P	High-temperature carbonization	1 M KOH	136	350	^15^
FeNi-NGE/NC	Thermal synthesis	0.1 M KOH	41.2	372	^23^
Fe–Ni–V	Chemical vapor deposition	1 M KOH	51	250	^3^
GFeNi 1:1.0	Sol–gel	1 M KOH	42	268	present work

To better understand the kinetics of the oxygen evolution reaction, the Tafel slope was derived from the LSV data using the equation (η = *a* + *b* log *j*).^68^ Where η is the overpotential, *a* is the intercept related to the exchange current density (*j*_0_), and *b* is the Tafel slope. As shown in Figure 6d, the Tafel slopes were 391 mV dec^–1^ for G and 68 mV dec^–1^ for GFeNi 1:1.0 (Table S1 of Supporting Information). The exchange current density for GFeNi 1:1.0 was calculated to be 4.06 × 1 × 10^–8^ A cm^–2^. These results from LSV and Tafel slope analyses indicate that the GFeNi 1:1.0 catalyst exhibits good OER kinetics compared to the other compositions tested. The superior OER catalytic performance of this GFeNi 1:1.0 material is associated with the synergistic effect between morphology and the presence of catalytically active surface sites. It could be justified by the thermodynamic effect. However, the plane (111) is more favorable for catalytic redox reactions than the (200) plane. This behavior is attributed to the large atomic number per unit area, which leads to the enhancement of the oxygen kinetic reactions by providing more catalytic sites. This finding was also observed by Khan et al.,^10^ when reporting the application of NiFe-based high-entropy alloys for the oxygen and hydrogen evolution reactions.

Aside from the morphology described in Figure 2 with a homogeneous distribution of FeNi nanoparticles in the graphene flakes network, determining the specific surface area is crucial (Figure 7), as it indicates the amount of surface area available for the processes or chemical reactions occurring on the material’s surface.^69^ It is evident that the gas adsorption process increases with rising relative pressure and decreases with falling relative pressure, indicating desorption. The shape of these curves corresponds to adsorption type IV with associated specific surface areas of 99.56, 163.62, and 121.61 m^2^/g for GFeNi 1:0.5, GFeNi 1:0.5, and GFeNi 1:0.5, respectively. This large specific surface area indicates great availability of active sites for the OER, favoring the occurrence of the electrochemical reaction of oxygen release. Furthermore, the results also indicate the presence of particles with a size ranging from 2 to 50 nm (Figure 7b) and an average pore diameter of 5.64 nm of the GFeNi 1:1.5 catalyst was observed (calculated using the Barrett–Joyner–Halenda technique), suggesting that the material has a mesoporous structure. In addition, the pore volumes of the electrocatalysts were 0.243, 0.546, and 0.426 cm g^–1^, for GFeNi 1:0.5, GFeNi 1:1.0, and GFeNi 1:1.5, respectively. The GFeNi 1:1.5 catalyst demonstrated a higher specific surface area and total pore volume, which are important for catalysis applications. This is known to facilitate the diffusion of electroactive species into the material’s pores, thereby enhancing the electrocatalytic performance of the catalyst.^16^

**Figure 7:**
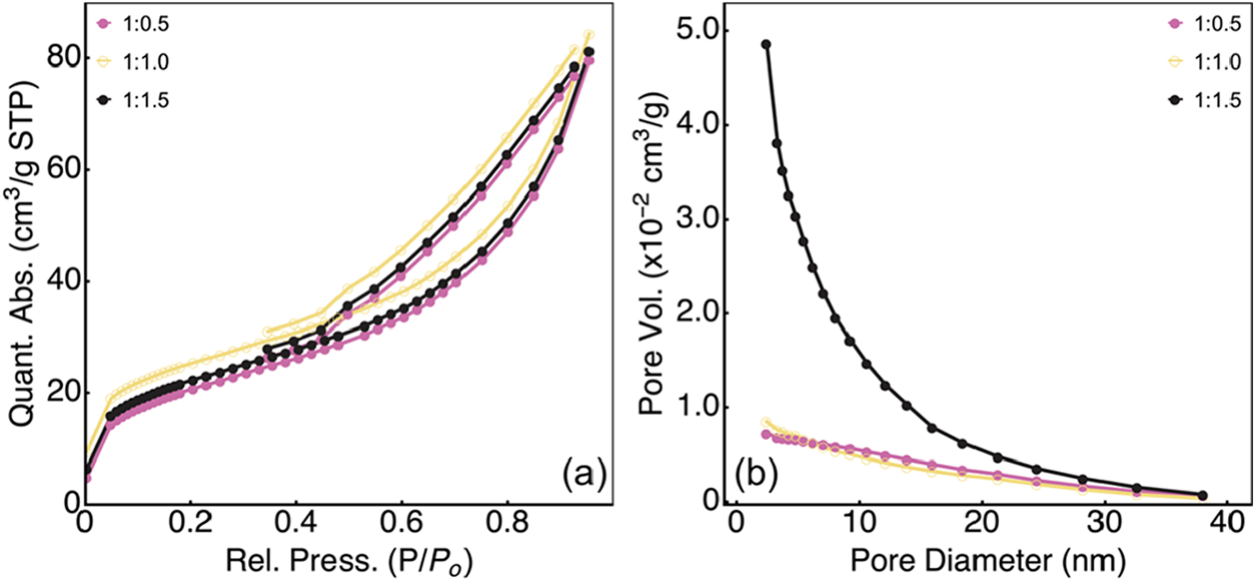
(a) Representative BET specific surface area plot for the studied GFeNi samples. (b) Particle size distribution for all studied GFeNi samples.

Since the electrocatalytic performance of a material is also related to its electrochemically active surface area, which is directly related to the double-layer capacitance (*C*_dl_), these two characteristics were determined in this study to better understand and explain the obtained results. The *C*_dl_ (mF) was evaluated by cyclic voltammetry and calculated from eq 6.^70,71^ The ECSA was determined according to the formula (eq 3).

2 $$J_{\mathrm{ia}}=\upsilon \times C_{\mathrm{dl}}$$

3 $$\mathrm{ECSA}=\frac{C_{dl}}{C_{s}}$$

where *J*_ia_ is the current density, υ the scan rate, and *C*_s_ the specific capacitance, which is 0.040 mF cm^–2^ for the transition metal-based catalysts in alkaline solution, as shown in Figure 6e.^22,72^ The *C*_dl_ and the ECSA of the GFeNi 1:1.0 catalyst was found to be 43.4 mF cm^–2^ and 1085 cm^2^, respectively. However, the higher OER catalytic performances cannot be attributed entirely to the increased ECSA, but also to the different species (oxide and/or hydroxide) formed on the catalyst surface during the electrochemical reaction, as reported in previous works.^28,29,62^ These results further support those relating to the specific surface area found by the BET technique.

Electrochemical impedance spectroscopy (EIS) was conducted in this work to investigate the type of mass transfer performed by the various electrocatalysts during the catalytic activity of the OER in a 1 M KOH electrolyte. The experiments were carried out across a frequency range of 100 kHz to 0.1 Hz, and the Nyquist curves were plotted as shown in Figure 6f. Figure 6g shows the electrical equivalent circuit used to fit the EIS data from Z view 3.2b software. Table S2 (Supporting Information) shows the values of the solution and charge transfer resistance, the double capacitance layer, and the correlation coefficient. The electrocatalysts G and GFeNi 1:0.5 demonstrated activity in a high-frequency region, followed by a large quasi-semicircle. Conversely, the electrocatalysts GFeNi 1:1.0 and GFeNi 1:1.5 exhibited activity in a high-frequency range, followed by a smaller semicircle. The observed small semicircles in the high-frequency domain may result from an increase in the concentration of Ni in the iron crystalline phase, which enhances the conductivity of the electrocatalyst. The different resistances contributing to the electrochemical process include the bulk electrolyte resistance (*R*_E_), the charge transfer resistance (*R*_CT_), and the equivalent series resistance (*R*_ES_). The relationship among these resistances is expressed by the following formula: *R*_ES_ = *R*_E_ + *R*_CT_. The *R*_CT_ values for electrocatalysts G, GFeNi 1:0.5, GFeNi 1:1.0, and GFeNi 1:1.5 were 75.8, 27.96, 2.99, and 3.92 Ω, respectively. We noticed that by increasing the nickel content in the GFeNi 1:1.5 catalyst, the charge transfer increases. This behavior could be due to the fact that high Ni content could lead to the accumulation of too many Ni positive charges, which reduces the active sites and hampers the charge transfer.^73^ The GFeNi 1:1.0 catalyst displayed the lowest *R*_CT_, indicating superior conductive behavior compared to catalysts G, GFeNi 1:0.5, and GFeNi 1:1.5. The GFeNi 1:1.0 catalyst was further characterized regarding its long-term stability (Figure 6h), showing quite stable behavior within 30 h of operation. It was observed that after 24 and 30 h of operation, the overpotential increased from 0.001 to 0.036%, respectively. The decrease in stability when increasing the time could be related to the oxidation of the main crystalline phases (NiFe, NiFe_2_O_4_, and graphene), leading to the formation of less-active species and therefore reducing the active sites.^74^

#### 3.5.1 Post-OER Studies

XRD analysis was performed on the substrate (CF) and the CF/GFeNi 1:1.0 catalyst before and after the OER experiments to better understand which phases are responsible for the OER performance of the electrocatalyst. The results are presented in Figure S2a (Supporting Information). It is observed in this figure that the main crystalline phases were graphite, NiFe, NiFe_2_O_4_, and FeOOH, which could be due to the graphite structure of the substrate, the deposition of the main phases FeNi and Ni_2_Fe_2_O_4_, and the oxidation of the catalyst after electrochemical tests. The catalyst exhibited the presence of the main crystalline phases of FeOOH (2θ = 76.2^o^) and FeNiOOH (2θ = 63.5^o^), with the main Miller indices (032) (JCPDS no 00-014-0556) and (110) (JCPDS No 96-100-9074), respectively.^75^ The presence of these oxyhydroxides proves the oxidation of FeNi and NiFe_2_O_4_ during the oxidation of the aqueous FeNi and NiFe_2_O_4_ during the OER experiments.

Furthermore, FTIR spectroscopy was performed on the catalyst post-OER analysis, and the results are presented in Figure S2c. It has been seen that after the electrochemical analyses, the FTIR spectrum of the electrocatalyst exhibited the presence of new absorption peaks. The peaks observed at around 700 cm^–1^ are attributed to the stretching vibration of the metal–oxygen bond (M–OH or M–OOH) (M = Fe or Ni).^75^ The bands at 863 and 1045 cm^–1^ were assigned to the bending vibration of the OH mode in the FeOOH structure. The band observed at 1680 cm^–1^ corresponds to the H–O–H bending vibration.^76^ These vibrations observed in the FTIR spectrum prove the modification of the electrode surface after the electrochemical experiments conducted in the electrolyte KOH 1 M. These results support those found in the XRD results. The cyclic voltammetry tests were performed at different electrolysis times with electrocatalyst GFeNi 1:1.0. The results (Figure S2c) show an increase in the oxidation/reduction current when increasing the time. The augmentation of the current intensity implies that the amount of M^2+^, which could be oxidized to MOOH, has increased. This behavior of the electrocatalyst could be due to the oxidation of the main crystalline phase FeNi, NiFe_2_O_4_, during the oxygen evolution process. The increase of this oxidation peak with time confirms the formation of new oxidant species during the electrochemical analyses. Moreover, previous works reported that when Fe and Ni are used as electrocatalysts in the OER study, the NiOOH and FeOOH active phases are formed during the process and further contribute to enhancing the electrochemical performance of the electrodes.^65,77^ The electrochemical reactions involved during the process are described as follows^65^

4 $$M+2{\mathrm{OH}}^{-}\to M{\left(\mathrm{OH}\right)}_{2}+2e^{-}$$

5 $$\mathrm{MO}+{\mathrm{OH}}^{-}\to \mathrm{MOOH}+e^{-}$$

6 $$M{\left(\mathrm{OH}\right)}_{2}+{\mathrm{OH}}^{-}↔\mathrm{MOOH}+H_{2}O+e^{-}$$

### 3.6 Mechanistic Insights

As it has been reported that monovalent iron-based catalysts are neither sufficient nor effective for the catalytic activity of the oxygen evolution reaction,^10^ it is hypothesized that the graphene plays a crucial role in the electronic properties, promoting the formation of electroactive species on the catalyst surface, facilitating the creation of active adsorption sites, and enhancing the catalyst’s performance. Nevertheless, previous works have reported that transition metal catalysts, especially Fe and Ni catalysts, could facilitate the electron transfer process and stabilize the reactive intermediates, leading to the enhancement of the OER catalytic process.^78,79^ Based on the XRD results, it is suggested that the three crystalline phases, namely, graphene, FeNi, and NiFe_2_O_4_, work synergistically to improve the OER catalytic activity of the OER by not only modifying the surface groups present at the catalyst interface but also defining the morphology. Furthermore, according to the results from the Tafel slope, the GFeNi 1.1.0 electrocatalyst showed a value of 104 mV dec^–1^, and consequently, the rate-determining step of the OER mechanism was the first reaction (eq 4). Thus, based on these findings and Krasil’shchikov’s OER mechanism, a plausible mechanism for OER catalytic activity occurring on the electrode surface is proposed.^80^ First, the OER mechanism begins with the adsorption of hydroxide ions (OH^–^) on the catalyst surface (*) (eq 7). Second, the adsorbed hydroxyl ions undergo a series of intermediate reactions, leading to the formation of hydroxyl radicals (*OH) or adsorbed oxygen (*O) (eq 8). The presence of metal species contributes to stabilizing the intermediates formed during the catalytic process and also facilitates electron donation.^81^ Third, the peroxide radical (*OOH) is formed by a reaction between the adsorbed oxygen radical and hydroxyl ions (eq 9). Then, the peroxide radical is oxidized to molecular oxygen (*OO) (eq 10). Finally, the O_2_ gas is generated on the surface of the catalysts and subsequently released into the solution (eq 11).

7 ∗+OH−⇌O*H+e−⁣Tafel slope=120mV dec−1

8 O*H+OH−⇌O*+H2O+e−⁣Tafel slope=30mV dec−1

9 O*+OH−⇌O*OH+e−⁣Tafel slope=40mV dec−1

10 O*OH+OH−⇌O*O−+H2O⁣Tafel slope=30mVdec−1

11 O*O−⇌∗+O2+e−⁣Tafel slope=22or40mV dec−1

These reaction steps illustrate the multistep nature of the OER process and highlight the importance of surface-active species and electronic conductivity in facilitating efficient charge transfer. The synergistic interaction among the FeNi alloy, NiFe_2_O_4_ phase, and conductive graphene matrix not only enhances the formation and stabilization of key intermediates but also promotes rapid electron transport, collectively contributing to the observed high electrocatalytic performance of the GFeNi 1:1.0 catalyst.

## 4 Conclusions

The autocombustion sol–gel process was successfully developed for the synthesis of ferromagnetic FeNi alloy nanoparticles uniformly supported on a graphene flake network. This method enabled the formation of well-dispersed FeNi nanoparticles and resulted in a homogeneous, interconnected composite structure. XRD analysis confirmed the presence of three main crystalline phases, graphene, FeNi, and NiFe_2_O_4_—across all Fe:Ni ratios investigated, indicating structural stability and reproducibility of the synthesis approach. Among the compositions, the GFeNi 1:1.0 catalyst stood out, demonstrating the best electrochemical performance with a low oxygen overpotential of 268 mV at 10 mA cm^–2^ and excellent operational stability over 24 h. This enhancement is attributed to the synergistic interplay between the nanoscale morphology, high density of catalytically active hydroxide species on the surface, and the high surface area and porosity conferred by the graphene support. The formation of Ni­(OH)_2_/NiOOH and FeOOH species during electrochemical activation contributed to the availability of accessible active sites and facilitated rapid mass and charge transport. This work addresses a critical gap in the field by combining magnetic FeNi alloys with graphene via a scalable sol–gel synthesis route, demonstrating that such hybrid systems can achieve electrocatalytic performance comparable to noble-metal-based materials. The integration of magnetic functionality also opens new possibilities for catalyst activation or modulation via external magnetic fields, a topic that remains largely unexplored in the field of OER studies. Future efforts will focus on evaluating the role of magnetic stimuli in dynamic electrocatalysis, scaling up the synthesis process, and integrating the catalyst into full water-splitting devices for practical hydrogen production applications.

## Supplementary Material



## Notes

CV plots of the catalysts performed at different scan rates in 1.0 M KOH electrolyte for all studied samples. Tafel slopes results, the electrical characteristics of the electrocatalysts, and XRD, FTIR, and CV results post OER analyses were also presented (PDF)
