# Supplementary material for: Facile Sol–Gel Synthesis of Graphene-Supported FeNi Nanocatalysts for Enhanced Oxygen Evolution Reaction
Source: ACS Omega. 2025 Oct 15;10(42):50431–44. doi: 10.1021/acsomega.5c07875 (PMC12573171; doi:10.1021/acsomega.5c07875)
Supplement: Supplementary file 1 [file ao5c07875_si_001.pdf]

Facile Sol-Gel Synthesis of Graphene-Supported FeNi Nanocatalysts for Enhanced Oxygen Evolution Reaction

Romuald Teguia Doumbi[1,2*], João Medeiros Dantas Neto[1,2], Artur de Morais[1,2], Awilvhygon Misker Dantas Freitas[1,2], Felipe Bohn[2], Kaline Pagnan Furlan[3], Dachamir Hotza[4], Carlos Alberto Martínez-Huitle[5], Marcio Assolin Correa[1,2]

[1]Postgraduate Program in Materials Science and Engineering, Federal University of Rio Grande of Norte (UFRN), 59078-970 Natal, RN, Brazil

[2]Department of Physics, Federal University of Rio Grande of Norte (UFRN), 59078-900 Natal, RN, Brazil

[3]Institute for Applied Materials – Ceramic Materials and Technologies (IAM-KWT), Karlsruhe Institute of Technology (KIT), 76131 Karlsruhe, Germany

[4]Postgraduate Program in Materials Science and Engineering (PGMAT), Federal University of Santa Catarina (UFSC), 88040-900 Florianópolis, SC, Brazil

[5]Renewable Energies and Environmental Sustainability Research Group, Institute of Chemistry, Federal University of Rio Grande do Norte (UFRN), Natal, RN, 59078-970, Brazil

Supplementary materials

First, the cyclic voltammetry (CV) tests were performed to activate the catalyst surface, as presented in Figure S1a-d. The CV experiments were conducted over several cycles and at different scan speeds. Interestingly, the peak current density values improved from the first cycle to the higher cycles, along with increasing scan speed. This may be due to catalyst activation by CV treatment. This figure shows the oxidation peak of hydroxide ions generating oxygen molecules at high potentials and current densities. This peak appears in all catalysts, but at different potentials. However, for catalysts with a high concentration of nickel, we note the emergence of a new peak at potentials of 1.3 and 1.4 V (Figures S1c,d). This peak appears at characteristic negative current densities and could be attributed to the redox reaction of Ni^{2+} ions to Ni^{3+} (Eq. 2-5). The width of this peak increases with the scan speed and is more accentuated on catalyst GFeNi 1:1.0. The broad area of the redox peak indicates the formation of catalytic species in the form of high-valent metal ions, which leads to a more efficient oxidation process than the parent metal ions. [1] Therefore, the catalyst GFeNi 1:1.0 is expected to be more effective in the OER than the other catalysts. The as-formed high-valent species act as strong oxidizing agents, further contributing to breaking the hydroxyl bonds in water molecules at higher rates, thereby further improving the catalytic OER activity of the electrocatalysts. [2,3]

$$Ni^{2+} \rightleftharpoons Ni^{3+} + e^- \quad (1)$$

$$NiOOH + e^- + H_2O \rightleftharpoons Ni(OH)_2 + HO^- \quad (2)$$

$$Fe^{2+} \rightleftharpoons Fe^{3+} + e- \quad (3)$$

$$FeOOH + e^- + H_2O \rightleftharpoons Fe(OH)_2 + HO^- \quad (4)$$

The Table S1 depicts the results obtained for the Tafel slopes for the studied samples. At the same time, Table S2 shows the EIS parameters of the electrocatalysts obtained from ZView software. Finally, Figure S2 brings to light the structural, FTIR, and CV curves comparison for the samples before and post OER experiments.

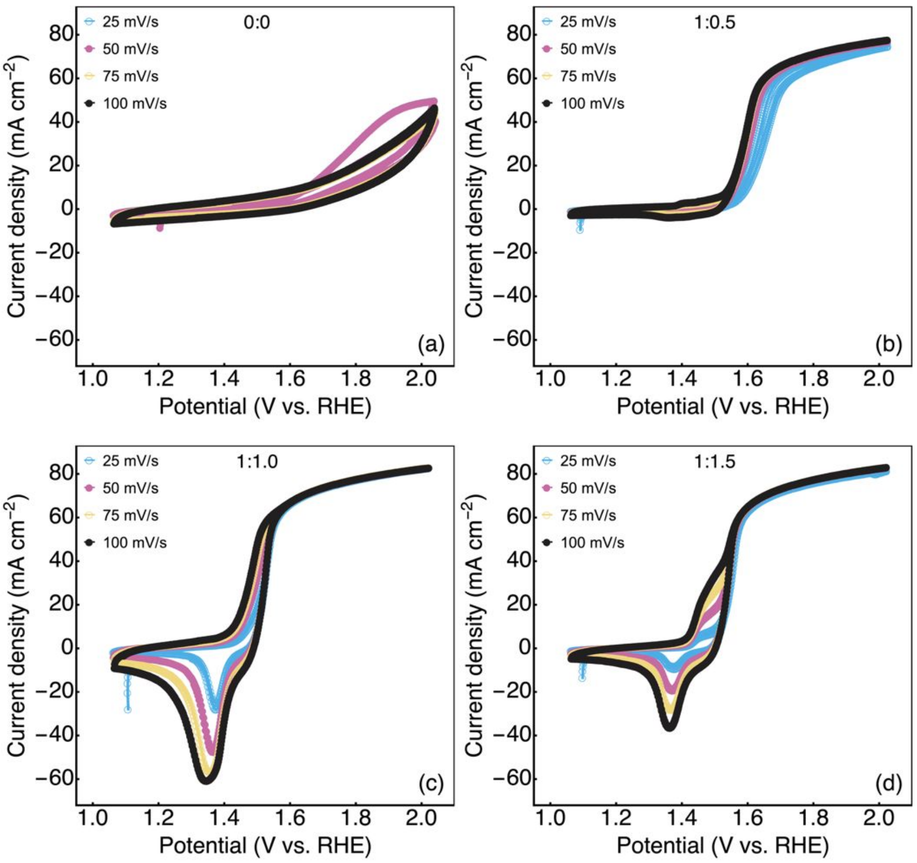

Figure S1. CV plots of the catalysts performed at different scan rates in 1.0 M KOH electrolyte for (a) Graphene sample (0:0). (b) 1:0.5 GFeNi sample. (c) 1:1.0 GFeNi sample. (d) 1:1.5 GFeNi sample.

Table S1. Tafel slopes of the G, GFeNi 1:0.5, GFeNi 1:1.0, and GFeNi 1:1.5 electrocatalysts

Catalysts	Potential range (V vs RHE)	R^2	Tafel slope (mV dec^{-1})
G	1.777-1.812	0.999	391
GFeNi 1:0.5	1.546-1.574	0.997	068
GFeNi 1:1.0	1.497-1.521	0.997	42
GFeNi 1:1.5	1.514-1.538	0.998	46

Table S2. Resumed the EIS parameters of the G, GFeNi 1:0.5, GFeNi 1:1.0, and GFeNi 1:1.5 electrocatalysts

Catalysts	R_s (Ω)	R_{ct} (Ω)	C_{dl} (F)	Errors (%)
G	8.44	75.8	0018206	8.569
GFeNi 1:0.5	10.35	27.96	0.00068	5.461
GFeNi 1:1.0	9.011	2.985	0.018803	3.211
GFeNi 1:1.5	8.978	3.921	0.0098469	4.203

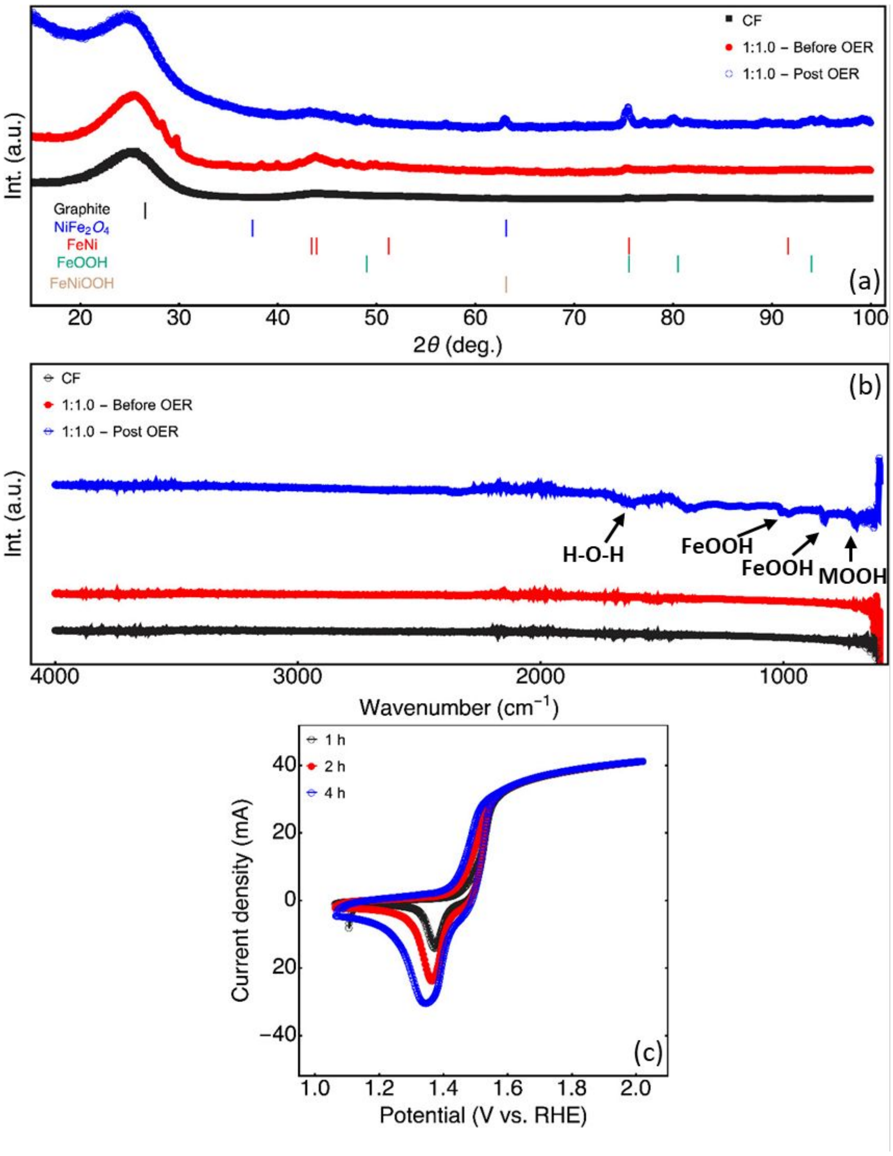

Figure S2. (a) XRD pattern of CF (carbon felt), GFeNi 1:1.0 before and Post OER experiments. (b) FTIR of CF (carbon felt), GFeNi 1:1.0 before and Post OER experiments. (c) CV curves of the GFeNi 1:1.0 electrocatalyst at different times.

References

(1) Ehsan, M. A.; Batool, R.; Hakeem, A. S.; Ali, S.; Nazar, M. F.; Ullah, Z. Controlled Deposition of Trimetallic Fe–Ni–V Oxides on Nickel Foam as High-Performance Electrocatalysts for Oxygen Evolution Reaction. *Int. J. Hydrogen Energy.* **2025**, *98*, 772–782.

(2) Amiri, M.; Golmohammadi, F.; Pure, A. E.; Safari, M.; Abbas, M. A. Engineering Iron–Nickel Nanostructures on the Surface of Functionalized Nitrogen-Doped Graphene Composite for High-Performance Supercapacitors. *J. Physics Chemistry Solids.* **2025**, *202,* 112699.

(3) Shah, S. A.; Ji, Z.; Shen, X.; Yue, X.; Zhu, G.; Xu, K.; Yuan, A.; Ullah, N.; Zhu, J.; Song, P.; Li, X. Thermal Synthesis of FeNi@Nitrogen-Doped Graphene Dispersed on Nitrogen-Doped Carbon Matrix as an Excellent Electrocatalyst for Oxygen Evolution Reaction. *ACS Appl. Energy Mater.* **2019**, *2* (6), 4075–4083.
